# Homophily and social influence as mechanisms of loneliness clustering in social networks

**DOI:** 10.1038/s41598-025-99057-x

**Published:** 2025-05-04

**Authors:** B. D. L. Châtel, R. Quax, G. Peeters, R. Corten, M. G. M. Olde Rikkert, V. V. Vasconcelos

**Affiliations:** 1 Department of Geriatric Medicine, Radboud University Medical Center, Nijmegen, The Netherlands; 2 Computational Science Lab, Informatics Institute, University of Amsterdam, Amsterdam, The Netherlands; 3 Radboudumc Alzheimer Center, Radboud University Medical Center, Nijmegen, The Netherlands; 4 Department of Sociology/ICS, Utrecht University, Utrecht, The Netherlands; 5 Department of Geriatric Medicine, Donders Institute for Brain, Cognition and Behaviour, Radboud University Medical Center, Nijmegen, The Netherlands; 6 Centre for Urban Mental Health, University of Amsterdam, Amsterdam, The Netherlands

**Keywords:** Public health, Computational science

## Abstract

Loneliness, a pervasive mental health concern, is often misconstrued as an individual pathology, limiting our understanding of social effects via peer-to-peer interactions. This study investigates how homophily (similarity-based connectivity) and induction (interacting mental states) contribute to loneliness clustering. Using a computational model, we simulate social network interactions via established induction frameworks: emotional, behavioral, and cognitive contagion. We map these pathways to fundamental processes of simple contagion, complex contagion, and self-activation, explaining how ideas and behaviors spread. Results show that high homophily is necessary for loneliness clustering, and the model recovers empirical findings of network clustering (a positive correlation of individuals’ mental states beyond direct neighbors) with extended “degrees of influence” across networks and setups. This universality of clustering across pathways renders the metric uninformative in screening causal mechanisms behind loneliness clustering. Fortunately, each inductive pathway displays distinct out-of-equilibrium dynamics, aiding in identifying real-world mechanisms. The study emphasizes the significant role of individuals’ social contexts in loneliness and calls for a shift from static to dynamic measurements in loneliness research. This shift will enhance the relevance of future research on evolutionary patterns in real-world social network data, leading to a more robust understanding of the mechanisms of loneliness.

## Introduction

Loneliness, a disparity between desired and actual social connectivity, has pervasive health and economic consequences. It is a complex multi-causal system outcome^1–3^ with numerous adverse (mental) health effects^1,4–6^ and adverse economic implications without known cost-effective interventions^7^. It is associated with an increased risk of developing clinical dementia^8^, depression^9^, and behaviors harmful to health^10,11^. Loneliness prevalence exhibits global variation, ranging from 11.6% in certain European nations to as high as 55% in Singapore^12^, while specific regions also display significant age-related disparities such as the Netherlands, with around 20% of individuals aged 55 and 62% of those aged 95 experiencing moderate to severe levels of loneliness^13^. The substantial economic cost of loneliness has significant implications. For instance, in the UK, loneliness incurs an estimated annual cost of 2.5 billion pounds to employers due to its diverse impacts, from employee health, reduced productivity, and caregiving responsibilities to increased voluntary staff turnover^14^.

While subjective loneliness is often viewed as an individual-centric issue^15^ distinct from social isolation (the objective absence or lack of sufficient social relationships), it is important to recognize that network-level factors do influence loneliness. Research suggests that lonely individuals tend to be connected with other lonely individuals, indicating the role of network influences in loneliness^16,17^. Lonely individuals are postulated to exert influence not only on their immediate friends (first-degree) but also indirectly on their friends’ friends (second-degree) and even the subsequent circle of connections (third-degree). This ripple effect reveals that loneliness holds a broader reach than individual experience, echoing through social connections. This concept, known as the “three degrees of influence,” was formulated by Christakis and Fowler in their research on the diffusion of traits in social networks, suggesting shared mechanisms with other social processes^16,18–20^. The clustering of loneliness can exacerbate its detrimental effects on physical and mental well-being^21^, as observed across different populations^16,17^. Recognizing the network-level dynamics of loneliness sheds light on its potential amplification and highlights its significance in public health and social research.

To devise effective interventions, understanding the underlying causes and their manifestations in social networks is paramount. The causal mechanisms underpinning the clustering of subjective loneliness are still not fully understood. Two theoretical social-science frameworks are posed to contribute to such clustering; homophily and induction^16^. Homophily refers to the tendency of individuals to form connections with others with similar attributes or characteristics^22,23^. Induction, on the other hand, refers to people influencing one another such that lonely individuals may contribute to the spread of loneliness in their immediate environment. In the context of loneliness, induction acts via three potential pathways^16^: cognitive, behavioral, and emotional contagion. The cognitive pathway suggests that loneliness arises from a mismatch between individuals’ social network expectations and their actual social experiences^24–26^. The behavioral pathway posits that lonely individuals may behave in ways that decrease relationship quality with others, thereby increasing the induction of loneliness in their surroundings^27,28^. The emotional contagion pathway is the phenomenon where people’s expressions and behaviors during interactions can influence and align their emotions. When individuals experience loneliness, their emotional state may be transmitted to others through emotional contagion, potentially spreading feelings of loneliness to those they interact with.^29,30^.

The observed clustering in various social phenomena beyond loneliness (e.g., smoking, obesity, and happiness^18–20^) could suggest the existence of general mechanisms that aptly describe individuals’ interactions. The process of information propagation can be described through what is now called simple contagion, a process of spreading that requires only one contact for transmission. Social networks with long-distance weak ties and small-world properties favor the spread of simple contagions^31^. Continuous processes, like opinion formation, can have similar properties, with the simplest model having opinions changing as a linear combination of neighbors’ opinions^32^. On the other hand, the nonlinear processes of behavioral adoption and technology innovation show stronger non-linearities. These can be represented by threshold responses, where multiple sources of reinforcement are required to induce change^33^, coined as complex contagion^34^. Complex contagions need strong ties and locally dense networks to propagate, but their relationship with clustering is nuanced, depending on the level of required reinforcement and whether they occur in isolation or in competition^35,36^. Similarly, nonlinear responses in opinion models can lead to different outcomes, depending on the network structure and the initial opinions^37,38^. Similar social contagion models have been applied to other phenomena, such as the spread of obesity, emphasizing the infectious nature of certain behaviors and states in social networks^39^. However, the computational mapping between simple and complex contagion processes and the proposed inductive pathways of loneliness and homophily is missing, while this can open the door for new interventions and inform new processes.

This paper seeks to fill the gap in understanding how homophily and induction, two key social frameworks, interact and contribute to forming loneliness clusters. Specifically, we evaluate if our computational agent-based model (ABM), simulating the asymmetric interactions between individuals following the theoretical framework posed in^16^, can replicate the empirical observation of the ’three degrees of influence,’ the cornerstone metric they used for measuring loneliness clustering. We follow this evaluation by investigating the sufficiency and necessity of the inductive pathways for inducing or upholding subjective loneliness clustering in the context of varying levels of homophily. Lastly, we identify the individual temporal dynamics of each inductive pathway under different levels of homophily, assess their mutual influences, and map the inductive pathways to either simple or complex contagion processes. Our findings can ultimately foster the generation of new behavioral hypotheses as the field of social contagion unveils fresh insights when aligned with emerging paradigms in social network analysis. By doing so, this research lays a solid foundation for devising and informing future strategies and interventions aimed at alleviating loneliness and fostering healthier interpersonal connections among individuals.

## Model

We consider a population of *N* individuals, each characterized by a dynamic value of social energy, representing an individual’s ongoing social activity engagement. This concept of social energy is derived from the Communicate Bond Belong (CBB)^40^ theory and serves as a simplifying assumption which proposes that humans have a fundamental need to belong and that social interaction is necessary to fulfill this need. The theory suggests that individuals possess a finite reserve of social energy, which is depleted or augmented by all forms of everyday talk and interaction, and that individuals regulate their social contact in a manner reflecting an underlying need state and the amount of social interaction in which they have previously engaged. In our model, we consider the accumulation of these interactions as the social energy of individual *i*, $e_i$, a continuous value between 0 and 1, which we model as a stochastic process. Lower social energy corresponds to greater loneliness. A directional complex network of social-energy-influence ties connects individuals. These ties carry the mechanisms responsible for changes in individuals’ social energy. We manipulate the social network structure to impose different degrees of homophily. Our analyses will evaluate whether each inductive pathway can autonomously maintain or generate clustering of loneliness. This assessment aims to gauge the self-sufficiency of these pathways in reproducing and maintaining the empirical findings of three degrees of influence. Subsequently, we will investigate various linear combinations of these pathways to explore their mutual influence. Below, we describe how we manipulate homophily, followed by the proposed computational implementation of the induction mechanisms. Additionally, we briefly describe how we measure the clustering of loneliness in this system. Further details are available in the Extended Methods in Supplementary Information A. Supplementary Table 1 is an overview of abbreviations, and Supplementary Table 2 justifies the default model parameters.

### Homophily and network initiation

We interpret homophily—an inherently dynamic phenomenon—as contributing to static network topology. This assumes distinct timescales governing induction and homophily; the latter evolves more gradually through relationships forming and dissociating based on traits that are hard or impossible to change, such as race, ethnicity, religion, education, or occupation^22^. This timescale separation simplifies the model by excluding dynamics of establishing or dissolving new connections among individuals.

We take homophily as a consistent distribution of specific homophilic traits within the network. This is operationalized by, first, assigning two labels associated with a homophilic trait (a priori unrelated to a tendency toward loneliness) to individuals, corresponding to two distinct but identical subpopulations of size *N*/2. Then, we adjust the network’s modularity^41^, *Q*, based on these fixed labels. Modularity over this trait quantifies the level of homophily present in the network, effectively contrasting relationships within subpopulations against those between subpopulations. We normalize the modularity such that a score of -1 signifies complete avoidance of one’s subpopulation (complete heterophily), a score of 1 signifies exclusive interaction within one’s subpopulation (complete homophily), and a score of 0 indicates random mixing between the two subpopulations.

To generate the networks of varying degrees of homophilic-trait modularity, we first initialize the population with extreme modularity ($Q = 1$). We create two identical, unconnected subpopulations within the network, each defined solely by its homophilic-trait label. Subsequently, we rearrange connections from within the subpopulations to connections between the subpopulations, thereby influencing modularity. As the two subpopulations share an identical topology, we uniformly randomly select a relationship and locate its counterpart in the opposite subpopulation. We rearrange these pairs, transforming two homophilic connections into two heterophilic ones, maintaining the degree distribution. Given a sufficiently large network, we can traverse the entire spectrum of modularity levels ranging from 1 to -1 by repeating this process. When all relationships within the subpopulations are rearranged, we achieve the extreme value $Q = -1$, resulting in a fully bipartite network configuration where only between-subpopulation connections remain. For a visual illustration of this procedure, see Fig. 1 in Supplementary Information A.

To comprehend the feasibility of inductive processes in maintaining a cluster of low social energy that was formed through homophilic processes ($Q=1$), as well as the potential emergence of clustering within random ($Q=0$) or even heterophilic networks ($Q=-1$), in the main text, we focus on the case where the initial levels of social energy are completely correlated with the homophilic trait.

### State update

Over time, agents continuously update their energy levels by weighting the three sociopsychological induction processes proposed by^16^. Relative weights of each pathway range from 0 (i.e., the pathway does not influence the agents’ energy) to 1 (i.e., the pathway is solely responsible for changes in the agents’ energy) in a linear combination.

**Fig. 1:**
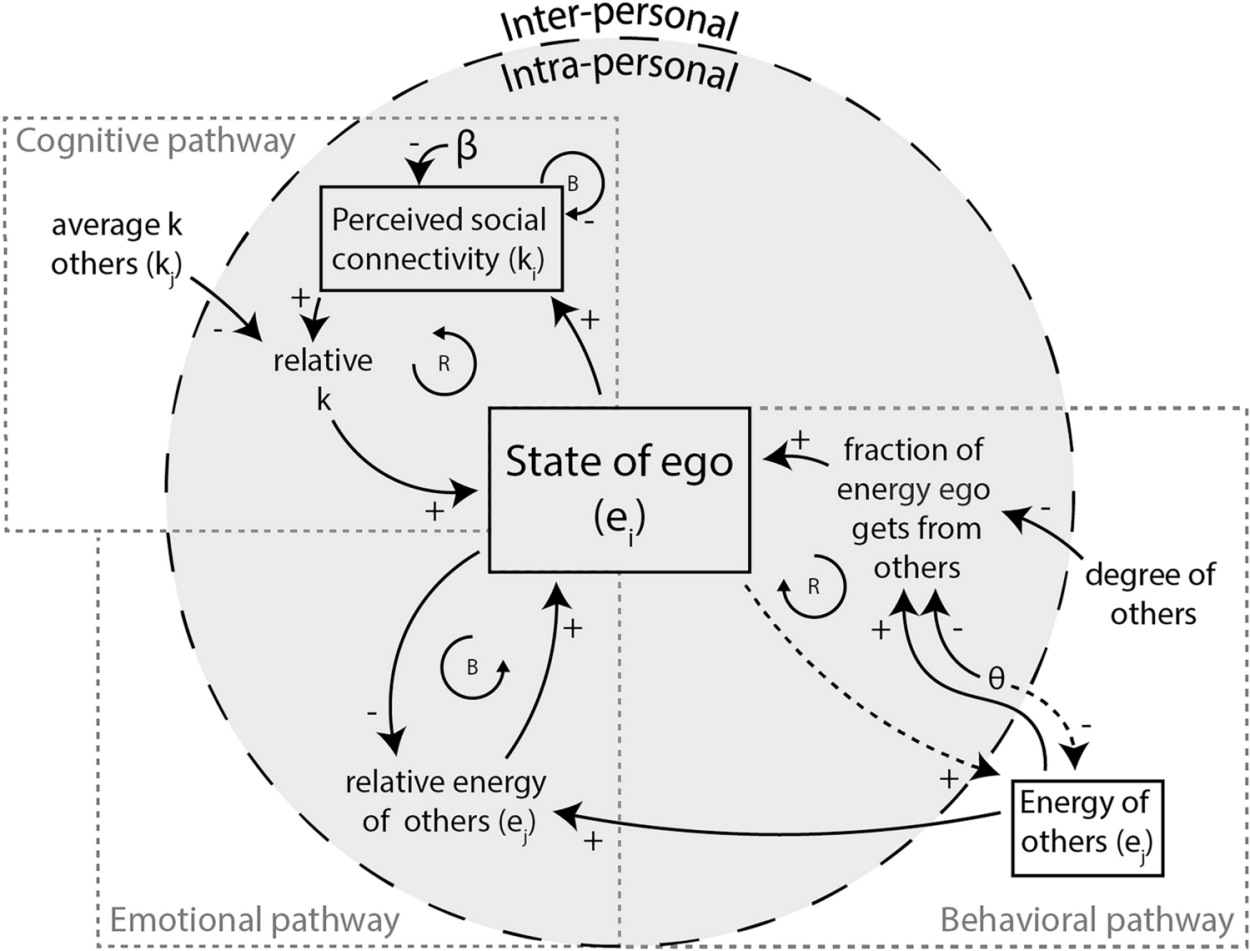
Causal loop diagram of social energy and connectivity dynamics in individuals within a social network. This diagram models the dynamic interplay of social energy (*e*) and connectivity (*k*) among individuals in a social network. Positive connections (+) indicate that a change in the influencing variable causes a change in the same direction in the influenced variable, while negative connections (-) denote an inverse effect. Solid arrows depict causal influences toward the ego, whereas dotted arrows represent effects toward other individuals (i.e., alters). Solid boxes enclose variables whose values accumulate over time, and variables without boxes are either static or auxiliary variables derived from other values. Reinforcing feedback loops are marked with “R,” and balancing feedback loops with “B.”.

We describe the different pathways from an individual perspective, though individuals are embedded in a social network. Let $a_{ij}$ be an adjacency matrix that identifies whether the ego individual *i* is connected to individual *j*, for which case $a_{ij}=1$, or not, $a_{ij}=0$. At each time point, *t*, individuals update their internal state, including their social energy, $e_i(t)$, based on different pathways. For convenience, we will ignore the time dependence whenever it is unnecessary to specify the time point. In Fig. 1, we present a Causal Loop Diagram (CLD) that summarizes the interactions of cognitive, behavioral, and emotional contagion pathways affecting individual social energy and perceived social connectivity within a social network. It distinguishes intrapersonal variables (within an individual) and interpersonal variables (between individuals).

#### Cognitive pathway

The cognitive pathway states that loneliness can arise from a discrepancy between the expectation of a given level of social connection and the perception of one’s connection to one’s social network. Perceived social connectivity, $k_i$, reflects how connected a person feels toward their social environment. This perception is affected by interactions with others and can change over time^16,24–26,42^, depending on the surrounding norms of social connection. Agents’ perceived social connectivity decay over time by a rate $\beta$, while social energy rebuilds it, resulting in

1 $$\begin{aligned} \frac{dk_i}{dt}=e_i - k_i \beta . \end{aligned}$$

The intuition here is that one needs to put social energy into upholding social relationships; without it, there will be a gradual reduction in (perceived) connectivity. This perceived social connectivity also feeds back into social energy. Specifically, the contribution of the cognitive pathway to change in social energy of an agent *i* is

2 $$\begin{aligned} f^\textrm{c}_i=k_i-\langle k_j \rangle _i, \end{aligned}$$

the difference between the perceived social connectivity and the mean perceived social connectivity of the agent in node *i*’s neighborhood, denoted by $\langle k_j \rangle _i=\frac{\sum _j a_{ji}k_j}{\sum _ja_{ji}}$ (i.e., a proxy for expectations based on its neighbors).

#### Behavioral pathway

Loneliness can lead individuals to act less trustingly and more hostile toward others, potentially harming relationships and perpetuating loneliness^5,27,28,43^. We model it via an interaction term between the self’s social energy and the neighboring nodes’ social energy contributions to the self, $\left\langle \frac{e_j-\theta }{d_j^+} \right\rangle _i$, with $d_j^+=\sum _la_{jl}$, such that nodes with fewer connections can allocate relatively more energy to a friend. Critically, we incorporate a threshold, $\theta$, to determine when others are lonely and may exhibit less trusting and more hostile behavior towards the self, reducing the self’s social energy. An individual with low social energy becomes less receptive to others, down-regulating the effect of others on the individual, as individuals may exhibit “less trusting and more hostile” behaviors as energy levels decline. We formalize the change in social energy of an agent *i* via the behavior pathway as

3 $$\begin{aligned} f^\textrm{b}_i=\left\langle \frac{e_j-\theta }{d_j^+} \right\rangle _i e_i. \end{aligned}$$

#### Emotional contagion pathway

Individuals may experience a convergence of emotions through (non)verbal communication^29,30^. Emotional contagion is the “tendency for the facial expressions, vocalizations, postures, and movements of interacting individuals to lead to a convergence of their emotions.”^16^ To model this process, we assume that agents converge towards the mean energy for the emotional contagion pathway upon interaction. The change in social energy of node *i* due to this process is given by the average social energy of the neighbors, $\langle e_j \rangle _i=\frac{\sum _ja_{ji}e_j}{\sum _ja_{ji}}$, relative to that of the self,

4 $$\begin{aligned} f^\textrm{e}_i=\langle e_j \rangle _i - e_i. \end{aligned}$$

#### Combining pathways

These exchanges during social interactions are aggregated according to the three inductive pathways. The pathways combine linearly to determine an individual’s social energy, resulting in the system of Stochastic Differential Equations:

5 $$\begin{aligned} dk_i&=(e_i - k_i \beta ) dt\end{aligned}$$

6 $$\begin{aligned} de_i&= \left( p_1f^{\textrm{c}}_i +p_2f^{\textrm{b}}_i+p_3f^{\textrm{e}}_i\right) dt+ \sigma e_i(1-e_i) dW_i, \end{aligned}$$

where $dW_i$ is a Wiener process of uncorrelated white noise, typically used to describe a collection of aggregated effects, for which the bias is capture by the drift terms described above. In our model, $p_1+p_2+p_3 \equiv 1$, where $p_1$, $p_2$, and $p_3$ represent the weights of the three induction pathways. To account for inherent uncertainties and randomness, a small diffusion term is added. Additionally, the diffusion function $e(1-e)$ constrains the social energy level between 0 and 1. To solve the stochastic differential equation we use an Euler-Maruyama scheme, with hyperparameter *h*, representing the step size for the numerical integration. We consider low noise values to maintain the dominant dynamics focused on the processes modeled and to guarantee some temporal variation across individuals in order to measure correlations over the network.

### Measuring clustering

**Fig. 2:**
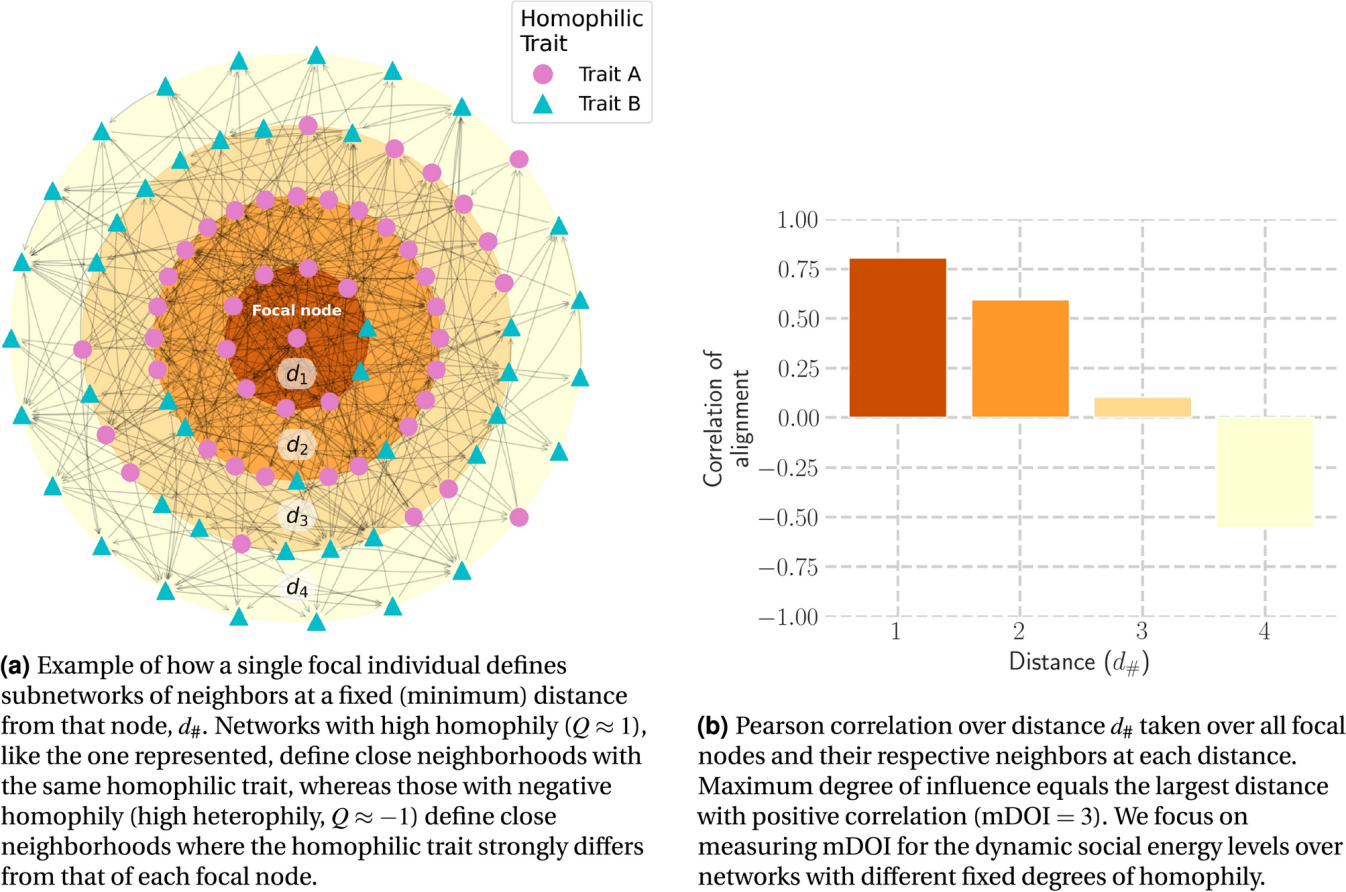
Example of how the maximum degree of influence (mDOI) is calculated.

To quantify loneliness clustering due to the inductive processes of loneliness, we tested whether our model could replicate empirical findings. We assessed whether any of the pathways replicates the “three degrees of influence” rule found in the body of work from Christakis and Fowler^16,18–20^. Our metric for measuring influence is the maximum degree of influence (mDOI), the largest network distance where the correlation of individuals self-organizing in the same trait is greater than zero^16,44^. Figure 2 illustrates this decline in correlation over distance, where the Pearson correlation is calculated over the social energy of each focal individual compared to their neighbors at different distances, corresponding to the circles of neighbors of different radii.

## Results

We examined the interactions between homophily and the three induction pathways to understand the causal mechanisms contributing to the clustering of loneliness in social networks. Following the framework proposed by Cacioppo et al.^16^, we assessed different configurations to investigate the reproducibility of the three degrees of influence observed empirically. We tested the maximum distance at which agents remained positively correlated or self-organized based on their social energy (i.e., the maximum degree of influence) over time and across varying levels of initial homophily. Next, we analyzed the community-level temporal dynamics of social energy to quantify the differences among the inductive pathways and identify the pathway with the greatest influence on the system. This comprehensive approach provides insights into the underlying mechanisms shaping loneliness clustering in social networks.

### Degrees of influence

**Fig. 3:**
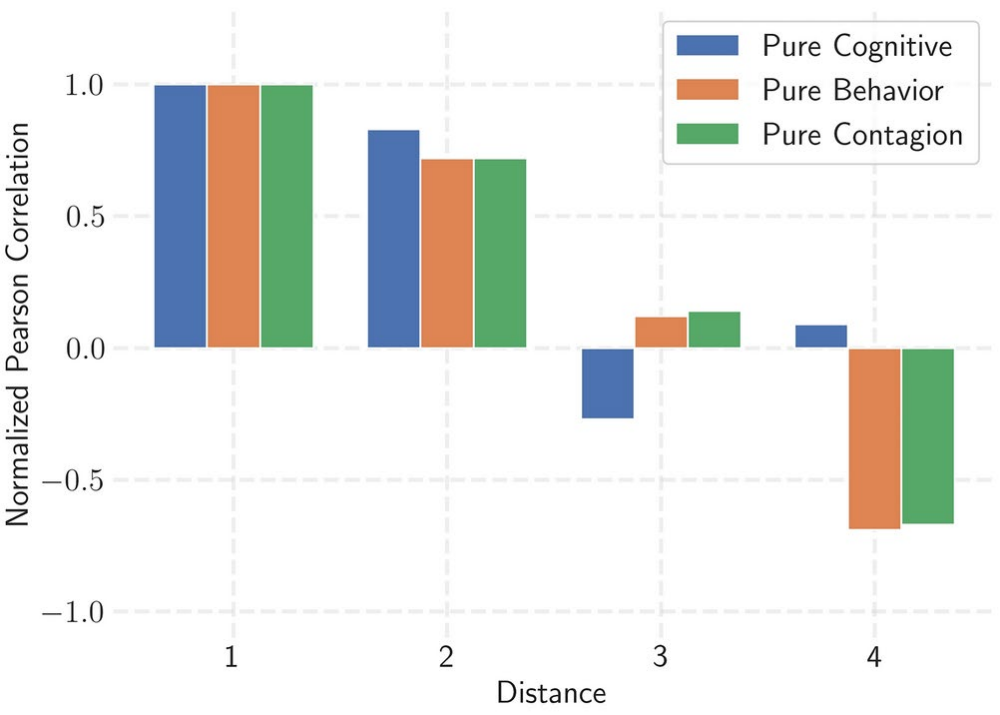
The correlation of self-organization on a social network ($Q=0.8$) across varying social distances for all three inductive pathways in isolation. Correlations are normalized on a distance of one. The cognitive pathway has a $\text {mDOI}=2$ for the social energy levels, while the behavioral and emotional contagion pathways have a $\text {mDOI}=3$, showing that the model is able to replicate the empirical findings of three degrees of influence. Values are taken for a network of size $N=1000$, measured at time 10.000, starting from a maximally clustered initial state, where half of the individuals have a high social energy level of 0.8 and the other half 0.2.

We assessed whether we could replicate the empirical finding of a degree of influence that extends beyond direct relationships. Figure 3 depicts the Pearson correlations over distance for each inductive pathway, normalized on a distance of one. This illustrates that the model can replicate between two and three degrees of influence on a highly homophilic network ($Q=0.8$).

**Fig. 4:**
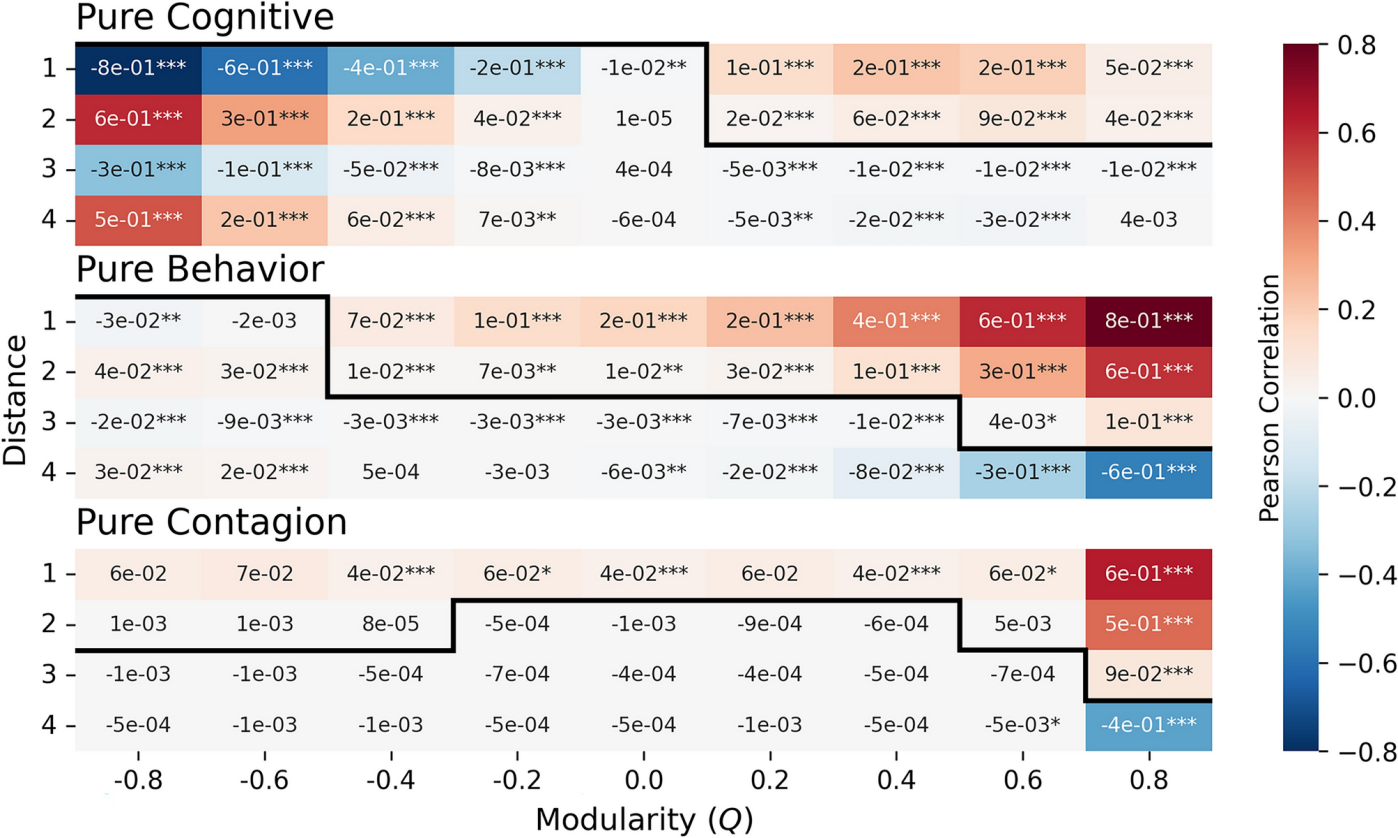
Heatmap depicting correlations across distances for each induction pathway at different levels of modularity. The black line indicates the maximum degree of influence (mDOI) across varying degrees of homophily and pathways. Each cell in the heatmap shows the average correlation derived from 20 simulations, and the number of asterisks indicates how many standard deviations the value is from zero, with three asterisks denoting a value at least three standard deviations away. The experimental setup is consistent with that described in Fig. 3. Refer to Supplementary Information B1 for a visualization of the correlation distributions.

We investigated the prevalence of a mDOI (maximum degree of influence) for different levels of homophily (modularity of the homophilic trait), *Q*, for each induction pathway, and after a long transient. We initialize the social energy levels such that, in the beginning, their Pearson correlation of social energies closely matches the level of homophily. We do so by matching one of the homophilic traits to a low initial social energy level and the other to a high initial social energy level. Figure 4 displays the social energy correlations over distance for each homophily level and induction pathway. The mDOI values are indicated by a dark black line, the point at which the correlations intersect the zero line. Our findings reveal the presence of positive correlations over distance, with mDOI values ranging from 1 to 3 across all pathways, even after a long transient. Across the three pathways, the long-term correlations tend to increase as homophily increases, particularly in homophilic networks ($Q>0$); zero or low homophily results in small correlations. When there is strong heterophily ($Q<0$), the Pearson correlation magnitudes for the Cognitive and Behavioral pathways maintain the initially imposed pattern of alternation, as relationships tend to align with the opposite subpopulation, whereas the Contagion pathway removes it. Additionally, although the mDOI values reach one or higher, the long-term Pearson correlations are not inherently high. In Supplementary Information B1, we show the full distribution of correlations over distance for each pathway across homophily levels. Supplementary Information B2 shows how the mDOI converges to the values shown. The degrees of influence for the Cognitive pathway converge quickly; the Behavioral pathway converges slowly for negative homophily levels; and the Contagion pathway exhibits a non-monotonic response, where the mDOI overshoots its equilibrium value. We provide evidence for the robustness of the results across network size and noise levels in Supplementary Information B3 and B4, respectively.

### Energy dynamics

**Fig. 5:**
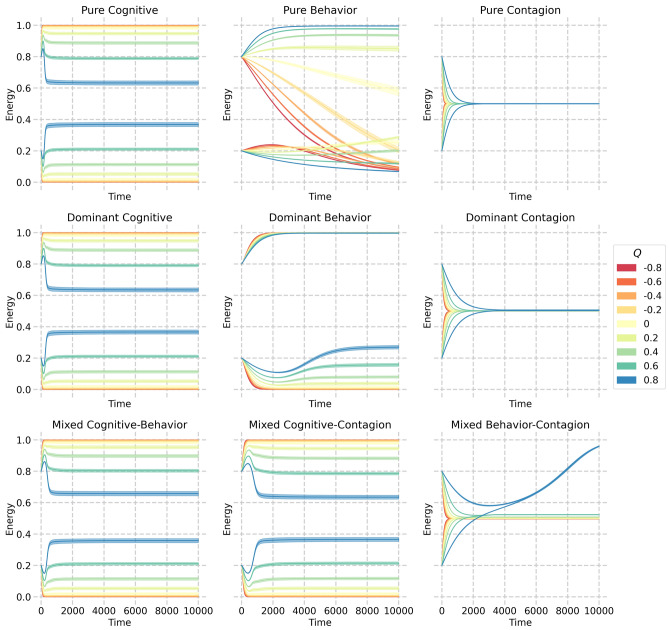
Mean energy dynamics per subpopulation over time for linear combinations of three inductive pathways. The line colors represent the starting modularity. Each panel presents the mean and standard error of the means based on 20 simulations. The top row of panels displays the purely active pathway, the middle row depicts a scenario where one pathway is dominant at 80% while the other two are at 10%, and the bottom row shows a 50/50 mix of two pathways.

Across the pathways, under positive levels of homophily, we recover up to three degrees of influence. Only for strongly heterophilic networks, the Contagion pathway is the only one reproducing clustering of loneliness, but the evidence for this type of network is weak^22^. Without additional microscopic calibration, this generalized result makes it challenging to differentiate between the various pathways and isolate the causal mechanisms behind loneliness clustering. Therefore, we analyzed the social energy dynamics of each inductive pathway, individually and mixed, by examining the mean energy dynamics per subpopulation over time. Figure 5 displays the dynamics over time both for each pathway in isolation, like in the previous analysis (top panel row), as well as for different mixes, including a mix where one pathway is dominant (middle panel row) and one in which two are equally present (bottom panel row).

Each pathway demonstrates distinct dynamics in isolation, which strongly reflect the mapping of the induction pathways to the type of contagion mechanism we established: self-activation, simple contagion, and complex contagion. The Cognition and Contagion pathways converge rapidly to equilibrium but with notable differences. The Cognition pathway, given its self-activating mechanisms, results in an outcome that largely depends on and ranks itself according to the initial states. The Contagion pathway, a simple contagion process, tends to converge towards the mean social energy. On the other hand, the Behavioral pathway exhibits more complex behavior, influenced by the network and its level of homophily, which determines its convergence pattern. This reflects the nuanced properties of complex contagion across different clustering and modularity measures.

The dominant pathway scenarios are such that one pathway is dominant at 80% weight in the social energy update process, while the remaining two are at 10%. In this dominant scenario, the dynamics of the Cognitive pathway remain unaffected by its combination with the other pathways. When the Behavior pathway is dominant, the Cognition route still drives dynamics, except for the initially energetic subpopulation, which moves towards full energy. When looking at the dominant Contagion pathway, the convergence rate is slowed.

Finally, we considered the combination of two equally active pathways. The cognitive pathway takes over the dynamics when equally mixed with the other two. When considering an equal mixture of the Contagion and Behavioral pathways, the Contagion pathway generally steers the system towards average energy values, with an exception occurring under conditions of high initial homophily ($Q=0.8$). In this scenario, a noteworthy threshold within the homophily level propels the system toward maximum energy. These dynamics arise due to the scaling of social energy between connected individuals and reveal the possible interplay between simple and complex contagion processes (refer to Supplementary Information A for detailed mathematical representation). We can better understand this phenomenon by examining the behavioral and contagion pathways individually (as depicted in the first row). At elevated homophily levels, the high-social-energy subpopulation swiftly converges to full energy, while the low-energy subpopulation experiences a more gradual convergence. The scaling of social energy among connected individuals leads to low-energy individuals undergoing minor energy changes, whereas those with high energy experience more pronounced shifts. The contagion pathway complements this process by driving the low-energy subpopulation towards convergence with the high-energy subpopulation, eventually leading to a fully converged high-energy population.

## Discussion

This study explores the interplay between the inductive pathways and homophily in clustering loneliness within social networks. First, our model replicates the three degrees of influence identified by Cacioppo et al.^16^ across inductive pathways, consistently for networks with positive homophily (i.e., high modularity in a homophilic trait). However, while this metric of “degree of influence” is a significant system feature, it does not provide insights for disentangling the different causal mechanisms, as each inductive pathway individually displays influence beyond direct connections under high levels of homophily. This universality where degrees of influence seem to emerge in transient states naturally, regardless of the underlying mechanisms or type of information being spread, is a finding supported by^44^, which we extend for continuous processes in directed networks. Furthermore, our findings indicate that the ability of inductive pathways to maintain loneliness clustering is contingent upon a positive level of homophily. This aligns with prior studies showing how homophily fosters the formation of clusters or subgroups within larger social networks^22^. In the long term, all pathways reduce high initial (anti-)correlations between social energy, except for the Cognitive pathway, which maintains anti-correlations for strongly heterophilic networks. However, the Behavioral and Contagion pathways further display a positive correlation where there initially was none or even a negative one ($Q<0$). These distinctions could be used for further discerning the true underlying mechanisms.

To further gain insight into the relationships between the inductive pathways, we analyzed their dynamics and their influence on one another. We are able to categorize the Cognition pathway as self-activating, the Behavior pathway as complex contagion, and the (Emotional) Contagion as simple contagion systems. This mapping can bridge the knowledge of a diverse range of disciplines in targeting loneliness.

The cognitive pathway results in a process that, to a large extent, is self-reinforced or suppressed at the individual level; these internal cognitive processes and beliefs act as self-activating factors in the experience of loneliness^5,45^ and are responsible for the creation of societal-level-reinforcing social norms regarding social connection^46^. External influence affects the threshold level of perceived connectivity that leads the individual to self-activate their energy level or self-suppress it. At the individual level, this pathway gives rise to a bistable system with two attractor states: a lonely state and a non-lonely state, which largely depends on the individuals’ initial states. Similar bistable dynamics are hypothesized to exist in other mental health issues, such as depression^47^. This self-activation likely contributes to the perception of loneliness as an individual pathology since the social environment regulates the internal state’s self-activation of -suppression values but not the state directly.

The behavior route is a complex contagion process^48,49^. In complex contagions, changes in individuals—usually relating to costly social behavior—require reinforcement from multiple contact sources, through a non-linear response to external information, usually in the form of a threshold response function^36^. The threshold we used to determine hostile behavior generates this critical mass of individuals within one’s social network needed for an individual to reduce (or increase) the social energy they put into others. The behavioral pathway is affected by the network topology as the more homophilic networks, compared to random networks, lead to a faster and more extensive spread of the behavior; this is in line with literature on complex contagion systems^36^. The convergence speed is affected by the changes in the level of homophily as wide bridges (i.e., many connections between communities) tend to speed up complex contagion as opposed to long bridges (i.e., few links formed with weak ties belonging to another community) having the opposite effect^50^.

The emotional contagion pathway aligns with the simple contagion (linear) model of information dissemination proposed by^32^, which describes how information spreads through social influence. Each interaction between individuals affects both parties in this process, leading to a mutual influence. For instance, facial expressions, vocalizations, postures, and movements can influence others in one’s immediate environment to become more lonely, leading to a convergence of emotions. Therefore, individuals will converge toward the mean energy of their surroundings. The results show that higher levels of homophily tend to slow down the spread of Emotional Contagion by limiting connections between different communities which is in line with the literature^48,51^. High levels of homophily confine the contagion to individual communities, while more random networks allow for rapid and widespread diffusion that saturates the entire network^49^.

Our modeling results point to the Cognition and Emotional Contagion pathways as the main determinants of dynamics over short and long periods. Each pathway exhibits unique energy dynamics, but the interaction between pathways complicates identifying individual trajectories and assessing causal contributions. To gain insights into the underlying processes contributing to loneliness clustering, future research could observe naturally perturbed real-world systems or investigate the formation of social networks based on subjective perceptions of loneliness, as explored by^17^, who inquired whether first-year college students form social networks based on subjective perceptions of loneliness.

Our study has several strengths. Firstly, we have developed a method that enables systematic sampling of homophily levels while maintaining the population’s degree distributions. We were able to use such an ABM to replicate the relevant clustering hypotheses on loneliness across pathways. Future research should investigate additional mappings of network topology and fixed homophily. These include manipulating triadic closure and modularity through community detection, which might highlight additional interactions between induction and homophily^52^. We focused exclusively on heterogeneity related to network connections and their influence on the evolution of dynamical states. Other forms of heterogeneity could be explored, such as different local environmental influences as shared environments among individuals may play a significant role in cluster formation in social networks^53,54^. Omitting this aspect could overemphasize the importance of induction and its interplay with homophily. Investigating the role of heterogeneity in future work is essential, but caution must be exercised as it could obscure structural understanding^55^. Lastly, since we take induction as given and do not model the process of network generation from an individual perspective, we cannot determine whether homophily alone provides sufficient and enough evidence of loneliness clustering.

Our theoretical model takes the three induction pathways as linear combinations of each other. Fit to data might require a non-linear schema and additional factors. Indeed, to broaden our understanding, future studies could use long-term data and in-depth interviews to investigate how different factors contribute to loneliness. This would provide a detailed dataset showing how loneliness spreads through social networks and shed light on how people’s similarities influence their connections. In this context, it might be relevant to consider situations where noise is more dominant in the dynamics and may even be modeled independently. Given that we found bistability at the individual level in some of the pathways, binarizing the data may be a solution once the other pathways are excluded. In that regard, discrete time (asynchonous) models may prove more tractable. These efforts could help us better grasp how loneliness works in real-life social situations. Ultimately, this could provide valuable insights that inform the refinement of our understanding, aiding in the generation of hypotheses for potential interventions. Moreover, future investigations could quantify inductive influences on an individual level, potentially leading to the development of targeted interventions aimed at strategically integrating individuals within social networks to counteract the clustering of loneliness. This approach aligns with the suggestion by^16^ that interventions focusing on peripheral individuals might help repair social connections and prevent network-wide unraveling.

### Conclusion

This study provides a nuanced understanding of how homophily and induction interact to form loneliness clusters within social networks. By employing a computational agent-based model, we effectively replicated the empirical observation of the “three degrees of influence” across different inductive pathways and varying levels of homophily. Our findings highlight the significant role of homophily in fostering loneliness clustering.

Each inductive pathway-cognitive, behavioral, and emotional contagion-displays unique dynamics, but their interactions complicate the identification of individual causal contributions. We found that the cognitive pathway is characterized by self-activation, whereas the behavioral and emotional pathways map to complex and simple contagions. These dynamics underscore the importance of considering individual and network-level factors in addressing loneliness, as these contagion types interact differently with varying network topologies.

Future research should further explore the interplay between homophily and inductive pathways, incorporating additional network topologies and forms of heterogeneity, such as environmental influences. This line of inquiry could pave the way for more targeted interventions to reduce loneliness by strategically integrating individuals within social networks. By focusing on dynamic measurements and real-world applications, this research can provide valuable insights for developing more effective strategies to mitigate the pervasive effects of loneliness on health and well-being.

## Supplementary Information

Supplementary Information.

## Data Availability

The simulation and analysis code supporting the findings of this study are openly available on GitHub. The repository, titled “Loneliness_Clustering”, can be accessed at the following URL:https://github.com/popoiopo/Loneliness_Clustering.
