# Supplementary material for: Homophily and social influence as mechanisms of loneliness clustering in social networks
Source: Sci Rep. 2025 May 4;15:15576. doi: 10.1038/s41598-025-99057-x (PMC12050302; doi:10.1038/s41598-025-99057-x)
Supplement: Supplementary file 1 — Supplementary Information. [file 41598_2025_99057_MOESM1_ESM.pdf]

Supplementary Information A: Extended Methods

Henceforth, we will use the following notations from Supplementary Table 1 to denote the concepts used in this paper.

Supplementary Table 1. Explanation of notations.

Notation	Explanation
i	Node/agent identification index.
j	Node/agent identification index of node initiating relationship towards i.
e_i	Energy level of agent i, an abstraction of the amount of energy one has to engage in social activity and used as a proxy for loneliness in our model.
k_i	Connectivity level of agent i, an abstraction of the how connected the agent feels to their social surroundings.
$\langle e_j \rangle$	Average energy taken over the incoming neighborhood nodes.
$\deg_i^+$	Out-degree, or number of outgoing relationships of node i.
$\deg_i^-$	In-degree, or number of incoming relationships of node i.
β	Connectivity decay rate.
ρ	Pearson correlation, or the assortativity, of the network for the energy property. Used to quantify induction effects by measuring the clustering of energy in the network.
Q	Modularity, the metric used to quantify homophily based on fixed labels of subpopulations.

Operationalisation of homophily

The model incorporates homophily by converging a network to a fixed state of modularity ($-1 \leq Q \leq 1$). First, two identical Barabasi-Albert subpopulations are created that only differ in initial energy. The resulting network exhibits a complete separation of relationships between these subpopulations, maximizing modularity. To achieve the desired modularity, the initial connections are indexed in each subpopulation and paired with identical counterparts in the other subpopulation. These pairs are then randomly rewired, introducing entropy into the system while preserving the degree distribution. This iterative process facilitates the network's progressive convergence towards modularity of -1, encompassing all intermediate values during the transition. Supplementary Figure 1 provides a visual representation of this process, while Supplementary Figure 2 provides a representation depicting the interconnectedness of the subpopulations over different initial modularities.

The resulting initial topology remains fixed throughout the simulation. Eliminating relationship formation, strengthening, and dissolution from the model reduces the need for additional nontrivial assumptions. Moreover, separating homophily from induction enables us to evaluate the dominant mechanism in the system and how these two mechanisms interact, as we can initiate networks with various modularity values.

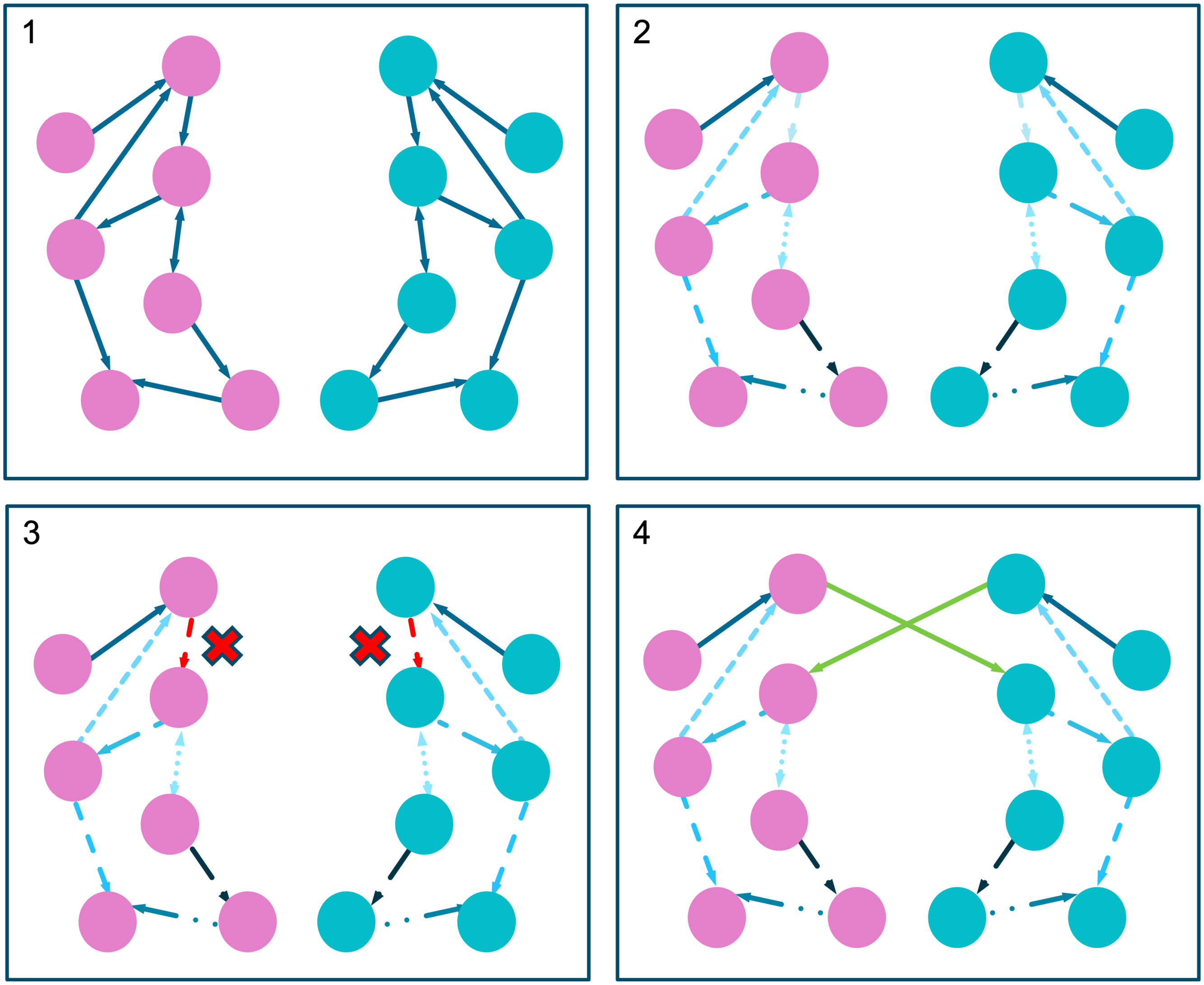

Supplementary Figure 1. Schematic representation of a single iteration of the rewiring method. Two identical graphs are used as subpopulations (N=500) with differing homophilic tags. Identical links are paired up (see pane 2), and a random pair is selected for link removal (i.e., the red relationships in pane 3). The removed link is then redirected to the other subpopulation (i.e., the green relationships in pane 4).

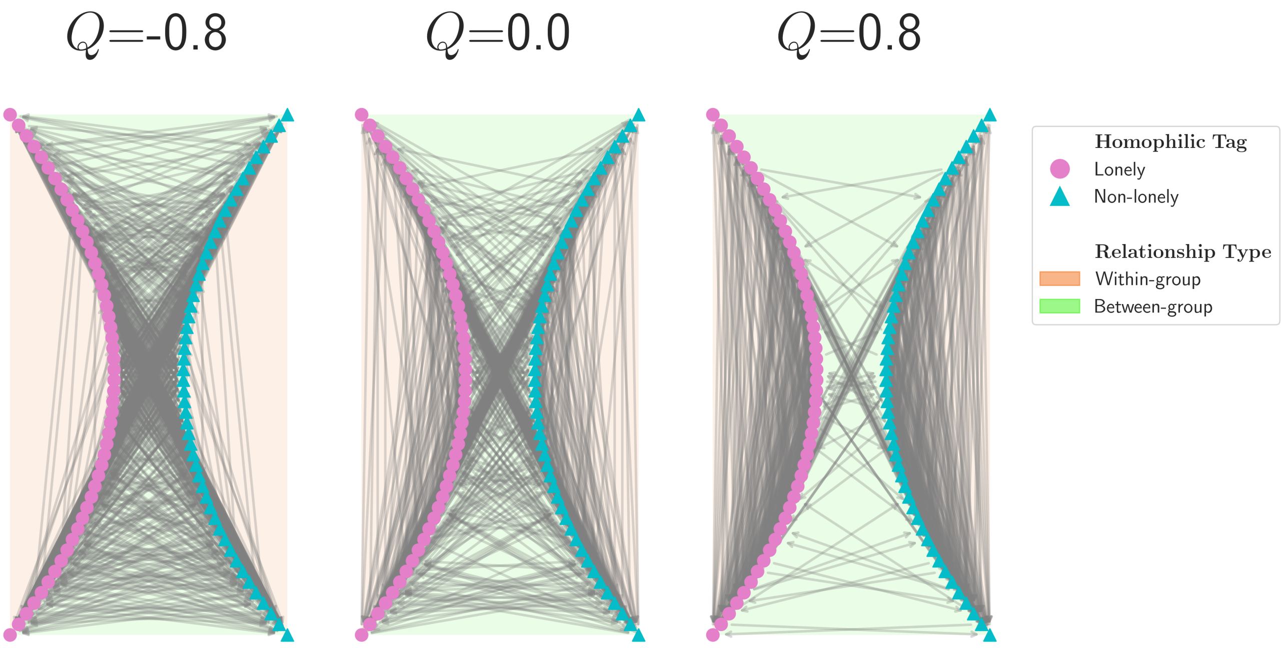

Supplementary Figure 2. The network representation illustrates negative ($Q = -0.8$), neutral ($Q = 0$), and positive ($Q = 0.8$) modularity. Within the diagram, the red-shaded areas represent connections within groups, indicating relationships among nodes with similar attributes. Conversely, the green-shaded areas represent connections between groups, denoting relationships among nodes with differing attributes. In addition to the shading, the nodes in the network exhibit different colors and shapes, signifying distinct homophilic tags.

Parameter values

Supplementary Table 2. An overview of parameter values used in the simulations.

**Parameter**	**Symbol**	**Value**	**Comment**
Population size	N	1000	Number of agents in the simulation.
Simulation duration	T	4000	Simulation duration in timesteps.
Number of edges per new node	m	11	Number of edges to attach from a new node to existing nodes in Barabasi-Albert algorithm.
Decay parameter	β	.5	Decay strength of social connectivity.
Modularity	Q	$-1 \leq Q \leq 1$	Metric of homophily measuring the interconnection between subpopulations in the initial network.

Supplementary Information B: Extended results

B1: Kernel Density Estimations of correlation distributions

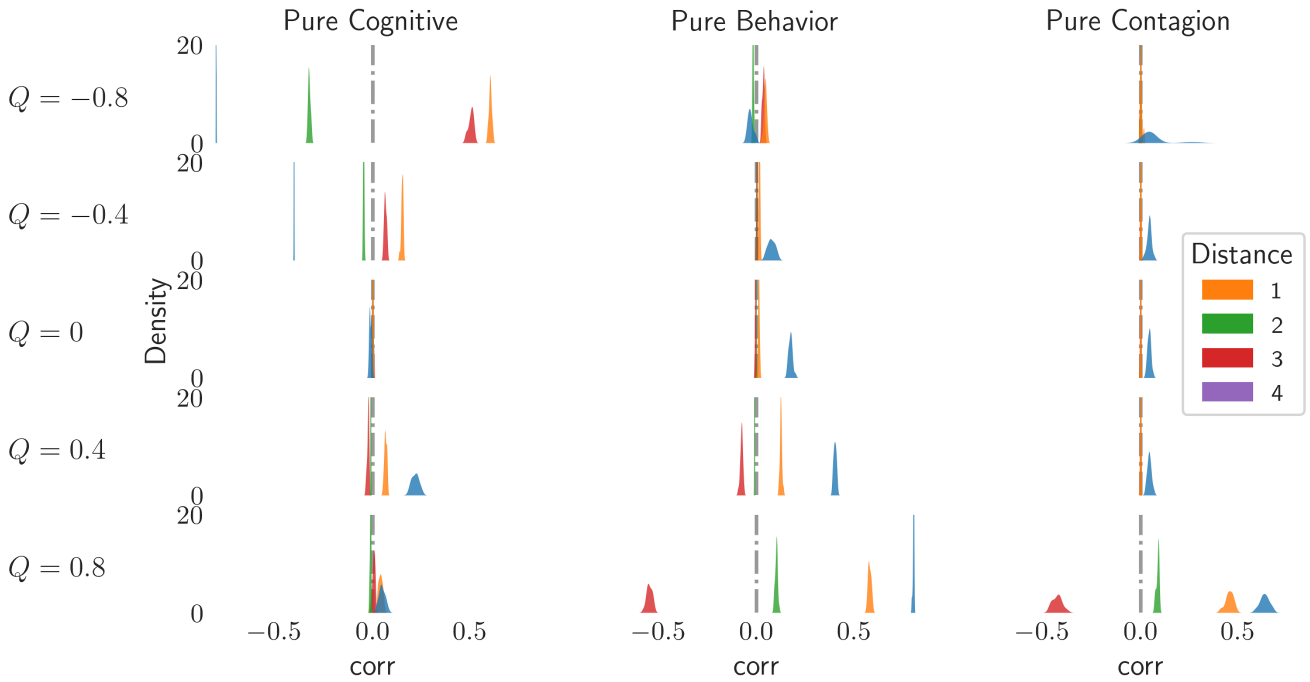

Supplementary Figure 3. Kernel Density Estimations of correlation distributions for each pathway over different modularities. Each distance is plotted separately and colored accordingly. We can see that distribution estimations are narrow and increase as Q increases.

B2: Degrees of influence over time

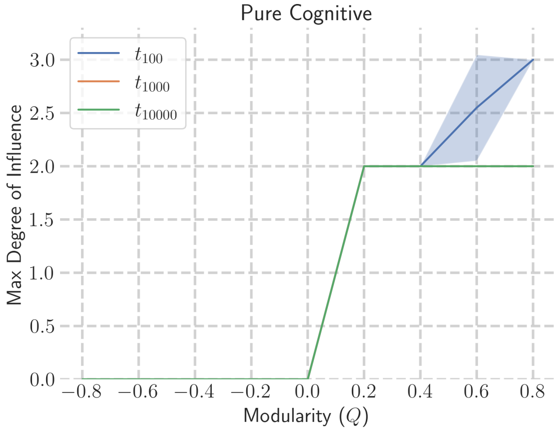

(a) Pure Cognitive

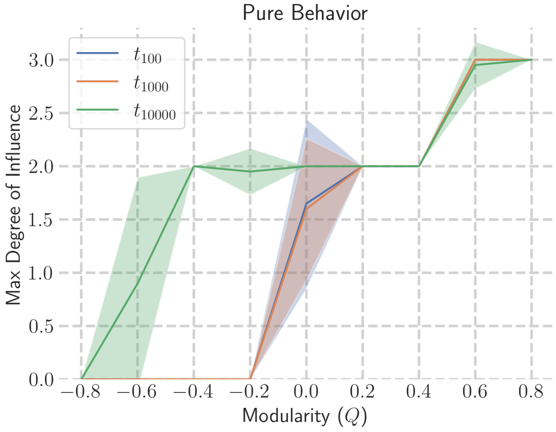

(b) Pure Behavior

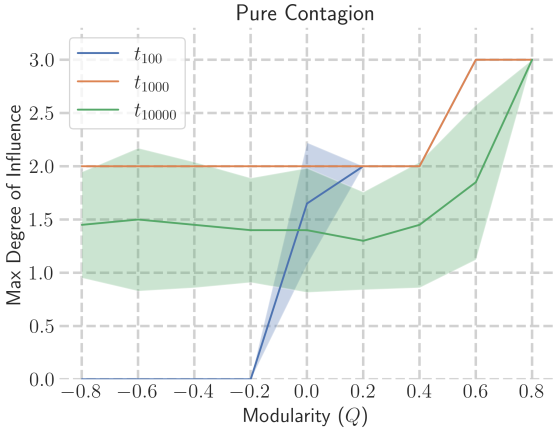

(c) Pure Contagion

Supplementary Figure 4. 4a), 4b), and 4c) illustrate the maximum degree of influence (mDOI) for different starting levels of homophily values and pathways over time (i.e., timestep 100, 1.000, and 10.000), providing insights into the stability of the clustering behavior in the network. The pure contagion and behavioral pathways display positive mDOI at negative modularities after 1000 timesteps, while the pure contagion pathway initially increases over simulation time while decreasing after convergence. Error bars indicate standard errors of the mean based on 20 simulations.

B3: Sensitivity of network size

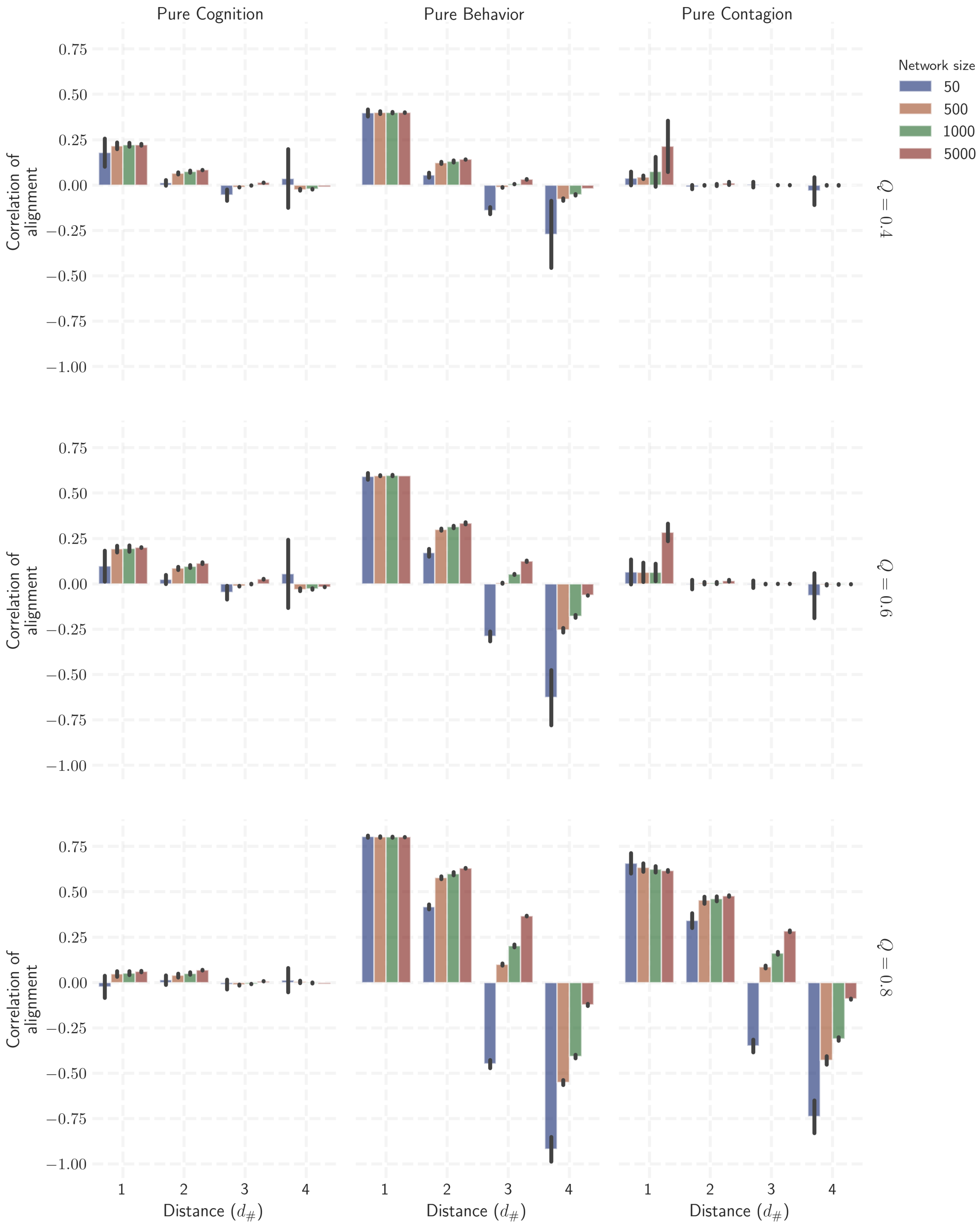

Supplementary Figure 5. Correlation over distance for different pathways (columns), over different modularities (rows). The colors represent network sizes, and the error bars represent standard deviations.

B4: Sensitivity of noise parameter

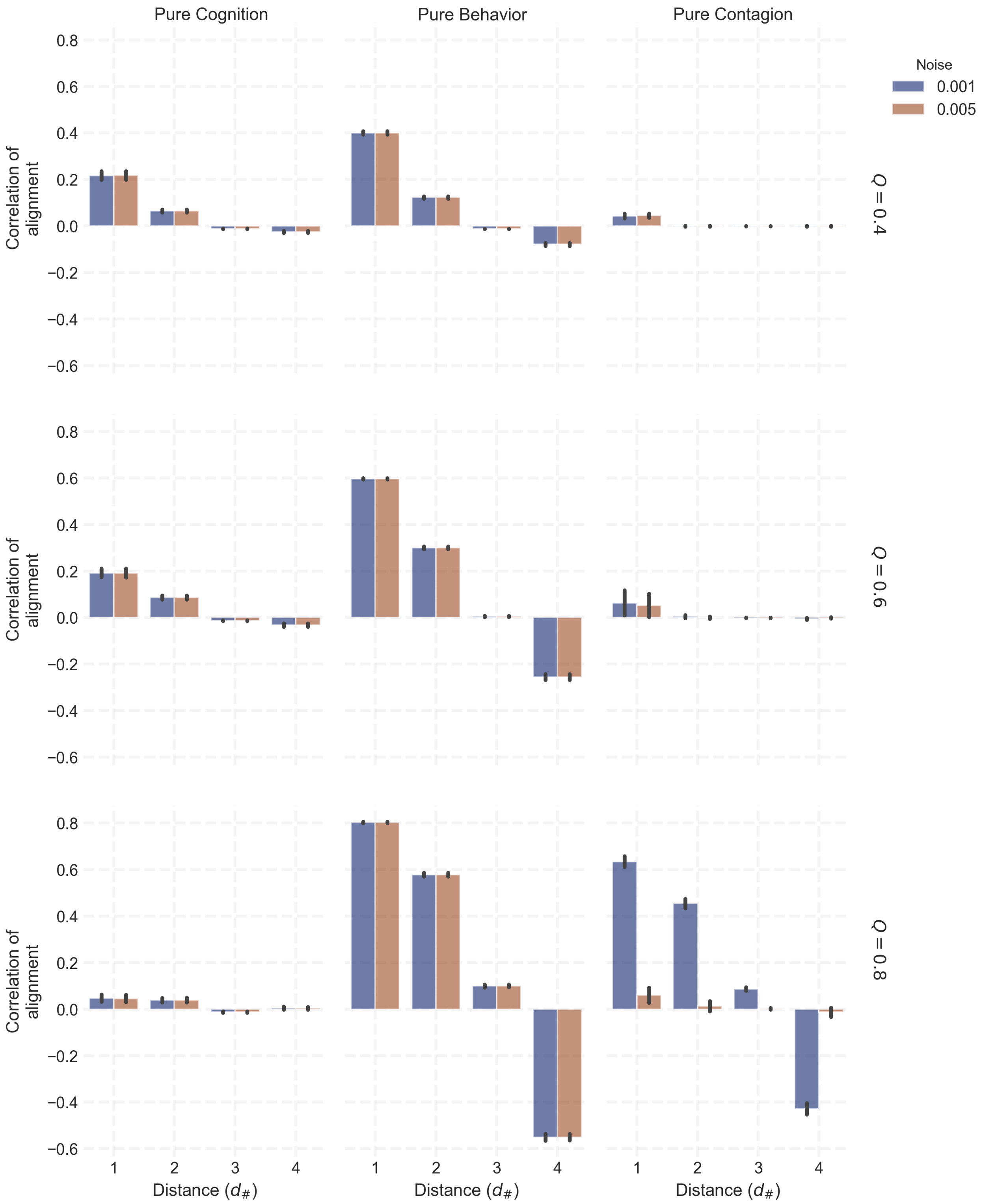

Supplementary Figure 6. Correlation over distance for different pathways (columns), over different modularities (rows). The colors represent the noise added, and the error bars represent standard deviations.
